# Dopamine terminals from the ventral tegmental area gate intrinsic inhibition in the prefrontal cortex

**DOI:** 10.14814/phy2.13198

**Published:** 2017-03-22

**Authors:** William C. Buchta, Stephen V. Mahler, Benjamin Harlan, Gary S. Aston‐Jones, Arthur C. Riegel

**Affiliations:** ^1^ Department of NeuroscienceMedical University of South CarolinaCharlestonSouth Carolina; ^2^ Neurobiology of Addiction Research CenterMedical University of South CarolinaCharlestonSouth Carolina; ^3^ Present address: Department of PharmacologyUniversity of Colorado School of MedicineAuroraColorado; ^4^ Present address: Department of Neurobiology and BehaviorUniversity of CaliforniaIrvineCalifornia; ^5^ Present address: Brain Health InstituteRutgers UniversityRutgers Biomedical and Health SciencesPiscatawayNew Jersey

**Keywords:** DREADDs, optogenetics, prefrontal cortex, ventral tegmental area

## Abstract

Spike frequency adaptation (SFA or accommodation) and calcium‐activated potassium channels that underlie after‐hyperpolarization potentials (AHP) regulate repetitive firing of neurons. Precisely how neuromodulators such as dopamine from the ventral tegmental area (VTA) regulate SFA and AHP (together referred to as *intrinsic inhibition*) in the prefrontal cortex (PFC) remains unclear. Using whole cell electrophysiology, we measured intrinsic inhibition in prelimbic (PL) layer 5 pyramidal cells of male adult rats. Results demonstrate that bath application of dopamine reduced intrinsic inhibition (EC_50_: 25.0 *μ*mol/L). This dopamine action was facilitated by coapplication of cocaine (1 *μ*mol/L), a blocker of dopamine reuptake. To evaluate VTA dopamine terminals in PFC slices, we transfected VTA dopamine cells of *TH::Cre* rats in vivo with Cre‐dependent AAVs to express channelrhodopsin‐2 (ChR2) or designer receptors exclusively activated by designer drugs (DREADDS). In PFC slices from these animals, stimulation of VTA terminals with either blue light to activate ChR2 or bath application of clozapine‐N‐oxide (CNO) to activate Gq‐DREADDs produced a similar reduction in intrinsic inhibition in PL neurons. Electrophysiological recordings from cells expressing retrograde fluorescent tracers showed that this plasticity occurs in PL neurons projecting to the accumbens core. Collectively, these data highlight an ability of VTA terminals to gate intrinsic inhibition in the PFC, and under appropriate circumstances, enhance PL neuronal firing. These cellular actions of dopamine may be important for dopamine‐dependent behaviors involving cocaine and cue‐reward associations within cortical–striatal circuits.

## Introduction

Reward learning involves burst firing of midbrain dopamine neurons in the ventral tegmental area (VTA) (Schultz, 1998, 2002). The resulting release of dopamine in terminal regions, including the prefrontal cortex (PFC), promotes learning and habit formation (Schultz et al., 1998; Schultz, 2002, 2006; Graybiel, 2008; Owesson‐White et al., 2008; Puig and Miller, 2012, 2015). Drugs of abuse influence dopaminergic mechanisms to promote habitual drug seeking, addiction, and relapse (Jentsch et al., 2000; Schultz, 2002; Ahn and Phillips, 2003; Heien et al., 2005; Chen et al., 2008; Thomas et al., 2008; Schultz, 2011).

The prelimbic (PL) area of the rodent medial PFC is anatomically and functionally distinct (Kalivas and Volkow, 2005). Tyrosine hydroxylase (TH) axons from the VTA contact PL cells that project to the accumbens (Carr et al., 1999). Within this circuit, the PL to nucleus accumbens core (NAcC) pathway is thought to form a motor subcircuit that contributes to initiating motivated behaviors, including drug‐seeking behaviors (Kalivas and McFarland, 2003; Kalivas, 2005; Lalumiere and Kalivas, 2008; Gipson et al., 2013; Stefanik et al., 2013). Moreover, glutamatergic projections in the PL‐NAcC pathway have been shown to be recruited in a dopamine‐dependent manner to drive reinstatement (McGlinchey et al., 2016). Therefore, a better understanding of how VTA dopamine regulates PL plasticity may provide insight into the mechanisms underlying drug‐seeking behavior.

Dopamine modulation of cortical plasticity involves D1‐like and D2‐like receptors, which activate diverse biochemical signaling cascades to alter synaptic transmission and neuronal excitability (for reviews, see Neve et al., 2004; Seamans and Yang, 2004; Beaulieu and Gainetdinov, 2011; Tritsch and Sabatini, 2012). Through these different effectors, dopamine alters the threshold for subsequent synaptic plasticity at excitatory synapses (Gurden et al., 2000; Seamans et al., 2001; Pawlak and Kerr, 2008; Zhang et al., 2009; Pawlak et al., 2010; Xu and Yao, 2010; Sheynikhovich et al., 2013; Ruan et al., 2014; Brzosko et al., 2015).

Considerable evidence suggests that modulation of intrinsic excitability, including changes in spike frequency adaptation (SFA or accommodation), plays an important role in neuronal plasticity and learning (Sah and Bekkers, 1996; Thompson et al., 1996; Cohen et al., 1999; Kramár et al., 2004; Fuenzalida et al., 2007; Cohen‐Matsliah et al., 2010; Zaitsev and Anwyl, 2012; Sehgal et al., 2013; Oh and Disterhoft, 2015). SFA is mediated in part by after‐hyperpolarization potentials (AHPs), resulting from the opening of calcium‐activated potassium channels (Madison and Nicoll, 1982, 1984; Lancaster and Nicoll, 1987; Stocker et al., 1999; Gu et al., 2005). SFA and the AHP (collectively referred to as *intrinsic inhibition*) function to reduce repetitive firing, allowing neurons to adapt to constant, low‐intensity excitation, and yet still respond to additional brief, intense depolarizing inputs (Benda and Herz, 2003; Benda et al., 2005; Prescott et al., 2006, 2008). Acute activation of dopamine receptors reduces intrinsic inhibition, transiently increasing neuronal excitability (Malenka and Nicoll, 1986; Pedarzani and Storm, 1993, 1995; Thurley et al., 2008; Yi et al., 2013). In multiple brain regions, changes in accommodation and the AHP correlate with learning and memory tasks (Coulter et al., 1989; Moyer et al., 1996; Thompson et al., 1996; Moyer et al., 2000; Zhang and Linden, 2003; Disterhoft and Oh, 2007; Santini et al., 2008; Matthews et al., 2009; Saar and Barkai, 2009; Mozzachiodi and Byrne, 2010; Sehgal et al., 2013). Whether direct stimulation of VTA terminals to release dopamine reduces accommodation and the AHP in the PL remains to be shown.

Here, we used patch clamp electrophysiology to measure accommodation and the AHP of layer 5 PL neurons during bath application of dopamine or stimulation of VTA terminals using optogenetics or chemogenetics. We observed that stimulation of VTA terminals reduced SFA and the AHP. This action was potentiated by cocaine, a dopamine reuptake inhibitor, and was observed in PL neurons that were verified to innervate the NAcC. Collectively, these data suggest that dopamine release from VTA terminals may gate intrinsic inhibition in the PL‐PFC by activating dopamine receptors to reduce SFA and the AHP. This mechanism may play a role in facilitating responses to salient stimuli predictive of rewards (Buchta and Riegel, 2015).

## Methods

All procedures were in accordance with the MUSC Institutional Animal Care and Use Committee. Adult (>P55) male Sprague Dawley (Charles River) and *TH::Cre* Long‐Evans rats (in house‐bred; initial breeding pairs provided by Deisseroth Laboratory, Stanford University) were used following acclimation to the vivarium (>7 days). Rats were single or double housed in a temperature‐ and humidity‐controlled vivarium under a reverse 12:12‐h light/dark cycle with free access to food and water. Prior to surgical procedures, which occurred in the light in a separate surgical suite, rats were anesthetized with a ketamine HCl/xylazine mixture (0.57/0.87 mg/kg, respectively, i.p.) followed by ketorolac (2.0 mg/kg, i.p.) and cefazolin (40 mg, i.p.).

### Viral transfection

*TH::Cre* ^+^ transgenic rats (P56–70) received bilateral injections of AAV2 (1–2 *μ*L; 10^12^ vg/mL) with the following constructs from the University of North Carolina Vector Core: EF1a‐DIO‐hChR2(H134R)‐EYFP (*n* = 6), hSyn‐DIO‐rM3D(Gs)‐mCherry (*n* = 4), or hSyn‐DIO‐hM3D(Gq)‐mCherry (*n* = 10) into VTA: AP −5.5 mm, ML ±0.8 mm, DV −8.15 mm (with respect to bregma). Four rats also received intracranial injections of fluorescent microspheres (150–300 nL, Lumafluor) into the NAcC (NAcC: AP 1.7 mm, ML ±1.6 mm, DV −6.8 mm). From four microsphere injected rats, locations into the NAcC were confirmed in acute brain slices fixed with 4% paraformaldehyde; one rat was excluded for missed placement. All injections were made through a glass micropipette (30–40 *μ*m diameter tip) using a Nanoinject II (Drummond Scientific). Pipettes remained in place for at least 5 min to minimize diffusion along the pipette track. Following 4–9 weeks of incubation, rats were sacrificed for immunohistochemistry or electrophysiology experiments.

### Immunohistochemistry

Immunohistochemistry was performed exactly as described previously (Mahler et al., 2014) on rats identically treated as those in physiology experiments, except prior to sacrifice they were perfused with 0.9% saline and 4% paraformaldehyde. After postfixing in 4% paraformaldehyde for 16 h and cryoprotection in 20% sucrose, brains were sliced in 0.1 mol/L phosphate buffer solution (PBS) with 1% sodium azide at 40 *μ*m. ChR2 and hM3D(Gq) receptor expression was visualized with immunohistochemistry for EYFP or mCherry tags. Dopamine cells were identified as tyrosine hydroxylase (TH) immunoreactive (Mahler et al., 2014). Sections were blocked for 2 h in 3% normal donkey serum and PBS with Triton X‐100 (PBST). Tissue was then incubated in normal donkey serum–PBST mixture for 16 h with primary antibodies, which included rabbit anti‐DsRed (mCherry tag; 1:1000; Clontech; 632496), chicken anti‐GFP (eYFP tag; 1:2000; Abcam; ab13970), and mouse anti‐TH (1:1000, Immunostar; 22941). After washing with PBST, sections were incubated 4 h at RT with donkey anti‐mouse 488 (TH; 1:500; Invitrogen; A21202), donkey anti‐rabbit 594 (mCherry; 1:500; Invitrogen; A21207), and/or donkey anti‐chicken 488 (GFP: 1:500, Jackson; 703545155). Slices were mounted and coverslipped with Citifluor mounting medium. All antibodies were previously validated for specificity, as described on the manufacturer's websites and the *Journal of Comparative Neurology* antibody database. ChR2 and hM3Dq expression was observed nearly exclusively in TH^+^ cells, as previously reported with this double‐floxed inverse open reading frame (DIO) vector in *TH::Cre* rats (Witten et al., 2011; Tye et al., 2012; Mahler et al., 2014; Zhang et al., 2015), and filled most of the anterior–posterior extent of VTA, and the most medial region of substantia nigra pars compacta (SNc).

### Electrophysiology

Rats were euthanized by rapid decapitation. The brain was gently removed, placed in ice‐cold artificial cerebrospinal fluid (aCSF) containing 126 mmol/L NaCl, 2.5 mmol/L KCl, 1.2 mmol/L MgCl_2_, 1.4 mmol/L NaH_2_PO_4_, 2.4 mmol/L CaCl_2_, 11 mmol/L glucose, 25 mmol/L NaHCO_3_, and 0.4 mmol/L ascorbate, and 0.01 mmol/L MK‐801, a NMDA receptor antagonist (Riegel and Williams, 2008; Williams et al., 2014). Brains were sectioned on a Leica VT1200S vibratome to make coronal slices (225–250 *μ*mol/L) containing the PFC (+3.0–3.7 AP from bregma). Slices were incubated (32°C) in aCSF with 0.01 mmol/L MK‐801 for at least 30 min prior to experimentation. Whole cell patch clamp experiments were performed on large pyramidal shaped cells in layer 5 prelimbic cortex, visualized using an Olympus BX51WI (Olympus America) equipped with gradient contrast infrared optics. Neurons expressing fluorescent microspheres were detected with collimated LED light (470 nm, ~0.7 mW) emitted through a 60X water immersion lens. The aCSF recording solution was identical to the incubation aCSF, except MK‐801 was excluded. Brain slices were perfused using a gravity‐fed system perfused at a flow rate of 2–3 mL per min and maintained at physiological temperature (~32°C). Recordings were made with MultiClamp700B (Molecular Devices). Patch electrodes (2–5 MΩ) were filled with internal solution containing 115 mmol/L K‐methylsulfate, 20 mmol/L NaCl, 1.5 mmol/L MgCl2, 10 mmol/L HEPES, 0.1 mmol/L EGTA, 2 mmol/L ATP sodium salt hydrate, and 0.3 mmol/L GTP sodium salt hydrate (pH 7.3; 270–275 mOsm). Membrane potentials for these pyramidal neurons typically ranged between −65 and −75 mV, with spike potentials above 0 mV, consistent with previous reports (Yang and Seamans, 1996a).

SFA was measured in current clamp configuration from a holding potential of −70 mV with input–output (I‐O) curves similar to previous reports (8 current injection steps; 0.1–1 nA; 800 msec; interstep interval 6 sec) (Madison and Nicoll, 1982; Lancaster and Nicoll, 1987; Aiken et al., 1995; Stocker et al., 1999; Velumian and Carlen, 1999; Shah et al., 2006). Unless otherwise noted, sample SFA traces illustrated in the figures were generated in response to 1000 pA current injections. Following the measurement of SFA, AHPs were recruited by a 60‐msec depolarizing step of varying amplitude (range: ~600–1500 pA) titrated to evoke five action potentials from a holding potential of −70 mV (once per minute, average of 2–3 sweeps, quantified as total area below the holding potential) (Coulter et al., 1989; Moyer et al., 2000; Gu et al., 2005). Pipette capacitance was neutralized. Series resistance was uncompensated, but monitored throughout experiments with brief hyperpolarizing steps. Experiments were discontinued if series resistance exceeded 30 MΩ or changed by >30%.

All drugs were applied via the perfusion media and complete exchange of the perfusion media in the recording chamber occurred in 1–2 min. Dopamine solutions were freshly prepared immediately prior to use and applied for 5–6 min. Intrinsic inhibition was then sampled in the continued presence of dopamine. Cocaine solutions were prepared daily. CNO was provided by the NIMH Chemical Synthesis and Drug Supply Program and Dr. Jurgen Wess (NIMH) with support by NIH‐NCI grant number X01 NS064882‐0. Cocaine was provided by NIDA Drug Supply Program. Dopamine was purchased from Sigma. All other compounds were purchased from Abcam/Ascent Scientific.

### Optogenetics

ChR2 stimulation occurred with purpose built collimated LED light (470 nm, ~0.7 mW) emitted through a 60X water immersion lens. For measurements of action potential firing during ChR2 photostimulation, VTA terminals were stimulated with blue light (473 nm). Each photostimulation episode consisted of eight trains of 10 msec light pulses applied at 17 Hz. This strategy was designed to mimic phasic dopamine neuron firing (Grace and Bunney, 1984; Schultz, 1998; Hyland et al., 2002). Firing in response to current injections (100–1000 pA) was evaluated during: (1) baseline (no light), (2) minute 1 (the first light exposure), and (3) minute 6 (the second light exposure). AHPs were measured during the intervening minutes 2–4 and again after the second light exposure. This paradigm was selected to facilitate comparison with responses to bath applied drug and to reduce the likelihood of phototoxicity. We observed no loss of SFA over a 25‐min recording period in similar whole cell recordings in slices from animals not transfected with ChR2 or from ChR2 transfected rat slices not exposed to photostimulation.

### Data analysis

All data (mean ± SEM) were analyzed in Axograph, Excel, and Prism. For comparisons of pre‐ and postdrug input–output curves within cells, all drugs compounds remained in the aCSF during postdrug measurements. Data were analyzed with two‐way ANOVAs matching both factors (cell and current injection), or paired and unpaired *t*‐tests for within and between cell comparisons. The dopamine concentration–effect curves were noncumulative, meaning each neuron was exposed to only a single concentration. Three‐parameter log (dose) versus response curves were used to fit dopamine dose–response curves, with EC_50_ values reported. For analysis of within cells correlation between our two metrics of intrinsic inhibition, AHP and action potential firing datasets were fit by linear regression, followed by a Pearson's correlation. Multiple comparison posttests were used as indicated. *P*s < 0.05 were considered significant.

## Results

### Dopamine reduces PL neuron intrinsic inhibition

**Figure 1:**
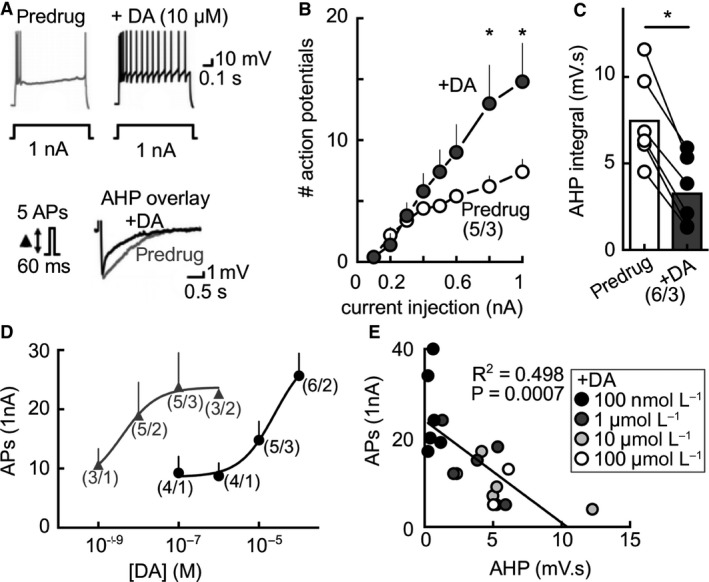
Dopamine reduces intrinsic inhibition in PL neurons. (A) Dopamine (DA, 10 *μ*mol/L; superfusion) reduces (B) SFA (two‐way ANOVA interaction: *F*_(7, 28)_ = 6.111, *P* = .0002; Sidak's post hoc) and the (C) AHP (*t*_(5)_ = 8.729, *P* = 0.0003). (D) Concentration–effect curve for dopamine on SFA (*black*; EC_50_: 25.0 *μ*mol/L). Coapplication of cocaine (1 *μ*mol/L) shifts the dopamine concentration–effect curve leftward (*gray*; EC_50_: 3.9 nmol/L). (E) Action potential firing and AHP measures show a significant negative correlation after various dopamine concentrations (linear regression; Pearson's correlation *F*_(1, 17)_ = 16.89, *P* = 0.0007). Values are mean ± SEM, *P < 0.05 compared to predrug. Numbers below represent cells/rats per group. SFA, spike frequency adaptation.

To investigate the effects of dopamine on intrinsic inhibition in the PFC, we made coronal brain slices containing medial PFC, and recorded from prelimbic (PL) layer 5 pyramidal cells identified by location, morphology, and electrophysiological criteria (membrane potentials at or below −60 mV, spike potentials above 0 mV). We assayed intrinsic inhibition using two interrelated metrics: SFA and AHP. SFA of action potential firing was measured in current clamp mode in response to depolarizing injections of current (0.1–1 nA steps of 800 msec) (Madison and Nicoll, 1984). The AHP was measured after a 60‐msec injection of depolarizing current, at an intensity adjusted to evoke five action potentials (Coulter et al., 1989; Moyer et al., 2000; Gu et al., 2005) (Fig. 1A). As anticipated, dopamine superfusion (10 *μ*mol/L) reduced intrinsic inhibition when spiking was triggered by current pulses of 1000 pA (Fig. 1A). This was reflected by an increased number of action potentials in the later period of the depolarization epoch (decreased SFA; Fig. 1A, B) and a decrease in the AHP (Fig. 1A, C). These effects occurred within 5–6 min of dopamine exposure. Dopamine (10 *μ*mol/L) induced a significant shift in spike frequency at current injections at 0.8 and 1 nA, with a trend toward a reduction at current injections between 0.4 and 0.8 nA (Fig. 1B). The AHP integral was on average significantly reduced by approximately half by 10 *μ*mol/L dopamine (Fig. 1C). The dopamine effect on SFA was dose dependent (EC_50_ + DA: 25.0 *μ*mol/L; Fig. 1D), and effective at nanomolar concentrations when coapplied with cocaine (1 *μ*mol/L), a blocker of dopamine reuptake (EC_50_ cocaine + DA: 3.9 nmol/L; Fig. 1D). Furthermore, additional analysis revealed a significant correlation between the effect of dopamine concentration on SFA and the AHP (Fig. 1E), suggesting dopamine may act on a signaling pathway regulating both the SFA and the AHP. These results support the possibility that dopamine may contribute to the inhibition of SFA and the AHP in the PL, similar to previous reports from PFC and hippocampal slices from younger rats (Malenka and Nicoll, 1986; Pedarzani and Storm, 1995; Thurley et al., 2008; Yi et al., 2013). As mentioned in the Methods section, these experiments were performed in slices incubated with the NMDA antagonist MK801. Likewise, we observed that application of NBQX to block AMPA receptors did not prevent or reverse our observed changes in SFA/AHP.

### Burst‐like activation of VTA^PL^ terminals reduces intrinsic inhibition in PL neurons

**Figure 2:**
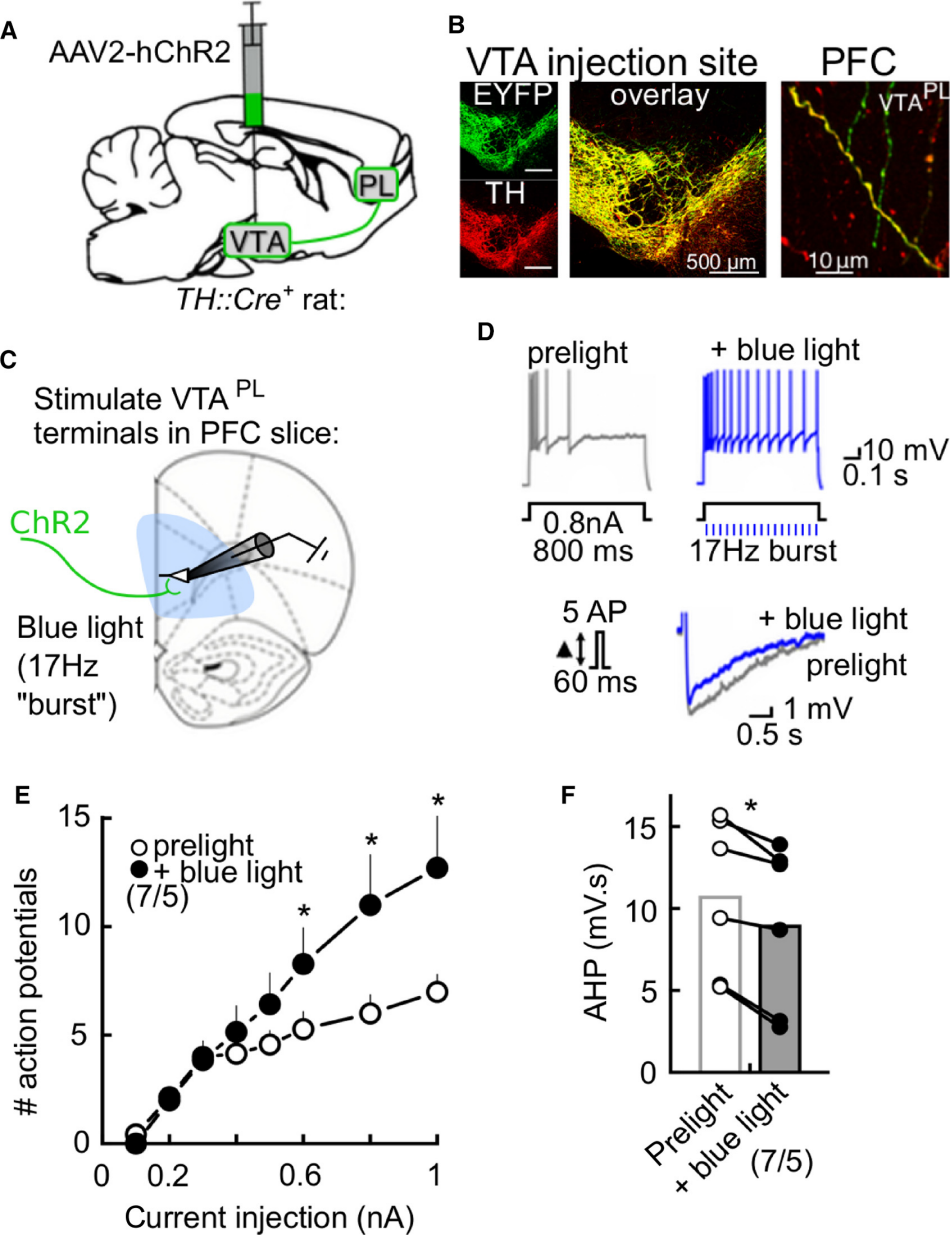
Burst‐like stimulation of VTA^PL^ terminals reduces intrinsic inhibition of PL neurons. (A) *TH::Cre* ^*+*^ rats received bilateral injections of AAV‐EF1a‐DIO‐hChR2(H134R)‐EYFP into the VTA. (B) At 4–9 weeks after transfection, we observed colocalization of TH (red) and ChR2 (green) in the VTA, and in fibers in the prelimbic cortex. (C) In similarly treated rats, acute PFC brain slices were made 4–9 weeks after transfection, and pyramidal neurons in the PL were recorded during burst‐like stimulation of ChR2‐VTA^PL^ terminals with blue light (473 nm; eight trains of fifteen 10 msec pulses at 17 Hz). Intrinsic inhibition was measured before and 5–6 min after initial exposure to blue light. (D) Example recording showing blue light illumination reduces SFA and AHP. Summary graph illustrating effects of blue light on (E) SFA (two‐way ANOVA interaction: *F*_(7, 42)_ = 7.674, *P* < 0.0001) and (F) the AHP (*t*_(5)_ = 5.111, *P* = 0.0037). Values are mean ± SEM. *P < 0.05. Numbers below represent cells/rats per group. PFC, prefrontal cortex; SFA, spike frequency adaptation.

In behaving animals, salient environmental stimuli can induce burst firing in VTA dopamine neurons (Schultz, 2002; Fiorillo et al., 2003; Tobler et al., 2005). Based on the results above, we wondered if dopamine release during burst‐like activation of VTA^PL^ terminals reduced intrinsic inhibition in PFC neurons. To evaluate this question, we intracranially injected Cre‐dependent viral vectors into transgenic rats expressing Cre recombinase under the tyrosine hydroxylase (TH) promoter (*TH::Cre* rats) (Witten et al., 2011; Mahler et al., 2014). A total of six *TH::Cre* ^*+*^ rats received injections of AAV‐EF1a‐DIO‐hChR2(H134R)‐EYFP (“AAV‐ChR2”) bilaterally into the VTA (Fig. 2A), allowing for temporal control of VTA^PL^ terminals with light. We performed immunohistochemical analysis for YFP expression in the VTA and PL in one rat at 4–9 weeks after injection, and observed robust expression of YFP nearly exclusively in TH^+^ cells in the VTA, similar to previous reports (Witten et al. 2011), as well as YFP expression in TH^+^ axon terminals in PL (VTA^PL^; Fig. 2B).

For electrophysiological experiments, the remaining five AAV‐injected *TH::Cre* ^*+*^ rats were sacrificed at 4–9 weeks after the injection to make acute PFC brain slices. Pyramidal cells in L5 PL‐PFC were recorded, and after obtaining baseline measurements of intrinsic inhibition, we stimulated ChR2‐containing VTA^PL^ fibers with burst‐like flashes of blue light (473 nm; 8 trains of 10 msec pulses at 17 Hz; delivered while measuring SFA; Fig. 2C) to mimic phasic dopamine neuron firing (Grace and Bunney, 1984; Schultz, 1998; Hyland et al., 2002). We observed that in three of seven PL cells recorded (~43%), 2–3 photostimulations were sufficient to reduce SFA and AHP (Fig. 2D). Averaging these responses across cells, the reductions in spike frequency at current injections 0.6–1 nA and the AHP in response to blue light was significant (Fig. 2E, F; *n* = 7 cells/5 rats).

### Activation of VTA^PL^ terminals reduces intrinsic inhibition in NAcC projecting PL neurons

**Figure 3:**
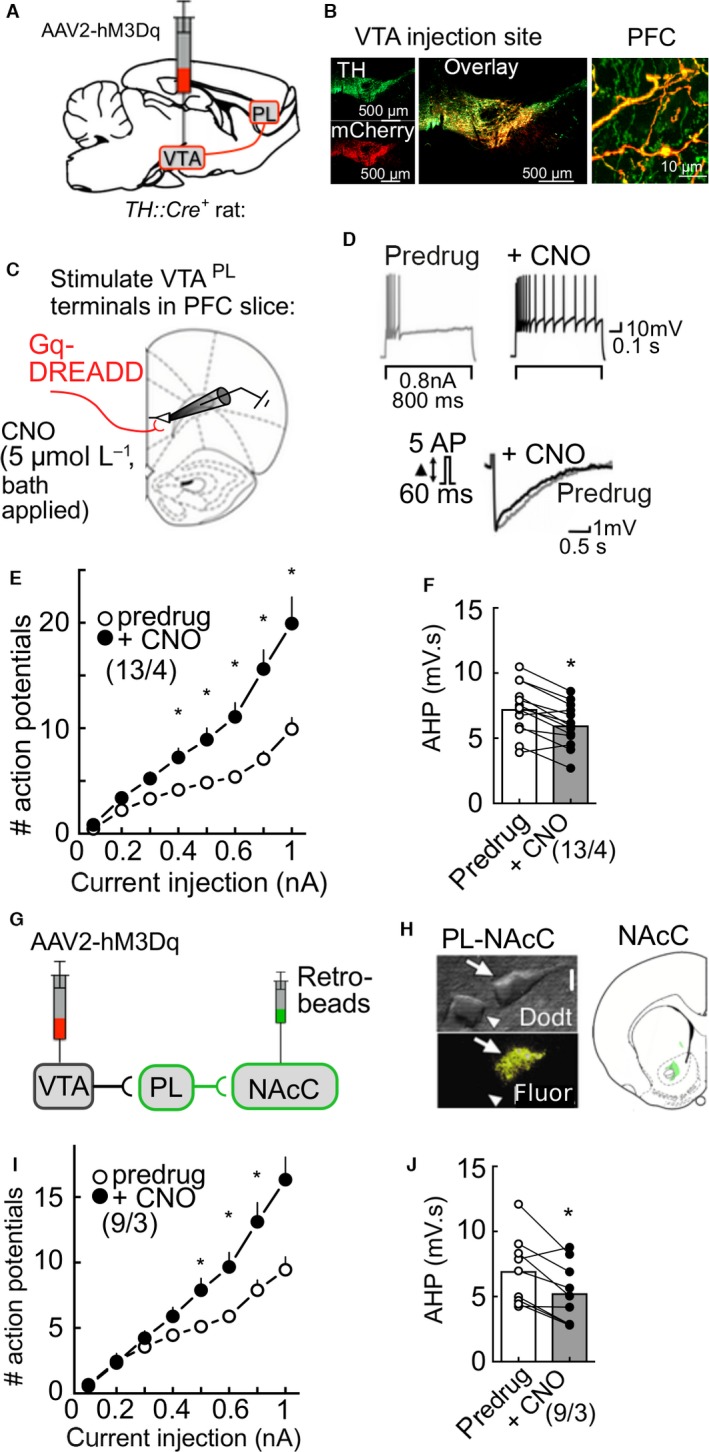
Intrinsic inhibition in PL‐NAcC projecting cells is regulated by DREADD stimulation of VTA^PL^ terminals. (A) *TH::Cre* ^*+*^ rats received bilateral injections of AAV2‐hSyn‐DIO‐hM3Dq‐mCherry into the VTA. (B) At 4–9 weeks after transfection, we confirmed colocalization of TH (green) and the mCherry tag (red) fused to G_q_‐coupled DREADDs in the VTA, as well as in fibers in the prelimbic cortex. (C) In similarly treated rats, acute PFC brain slices were made 4–9 weeks after transfection, and pyramidal neurons in the PL were recorded during stimulation of VTA^PL^ fibers with CNO (5 *μ*mol/L) applied via bath perfusion. (D) Sample traces of a PL pyramidal neuron showing reduction of SFA and AHP in response to CNO. (E) Summary graph illustrating CNO reduces SFA (two‐way ANOVA interaction: *F*_(7, 84)_ = 22.79, *P* < 0.0001; Sidak's post hoc). (F) Summary showing AHP responses from PL neurons before and after CNO (*t*_(12)_ = 5.056, *P* = 0.0003). (G) To record specifically from PL PFC neurons projecting to the NAcC (PL‐NAcC), rats received injections of retrogradely transported fluorescent beads into the NAcC and bilateral injections of AAV2‐hSyn‐DIO‐hM3Dq‐mCherry into the VTA. (H) *Left:* High‐power Dodt image from an acute PFC slice showing two cells in the recording chamber, one pseudocolored bead‐labeled PL‐NAcC neuron (arrow) and a nearby unlabeled cell (arrow head). *Right:* Low‐power confocal image showing example injection site of fluorescent beads in the NAcC. (I) Summary graph from PL‐NAcC neurons illustrating CNO reduces SFA (two‐way ANOVA interaction: *F*_(7, 56)_ = 9.195, *P* < 0.0001; Sidak's post hoc). (J) Summary showing AHP responses from PL‐NAcC neurons before and after CNO (*t*_(8)_ = 3.487, *P* = 0.0082). Values are mean ± SEM. *P < 0.05 compared to predrug. Numbers below represent cells/rats per group. PFC, prefrontal cortex; VTA, ventral tegmental area.

Given the selective responsiveness of some PL cells to optogenetic stimulation of VTA terminals, we used a chemogenetic strategy to achieve a more sustained activation of VTA fibers. We bilaterally transfected the VTA of 10 *TH::Cre* ^*+*^ rats with AAV‐hSyn‐DIO‐hM3D(Gq)‐mCherry and 4 *TH::Cre* ^*+*^ rats with AAV‐hSyn‐DIO‐hM3D(Gs)‐mCherry (Fig. 3A). After 4–9 weeks of incubation, we confirmed in three AAV‐hM3D(Gq) transfected rats that this procedure resulted in robust expression of mCherry‐tagged DREADDs in TH^+^ VTA cells (97% coexpression of TH in mCherry^+^ neurons), similar to previous reports (Mahler et al. 2014), as well as in TH^+^ axon terminals in the PFC (Fig. 3B).

We evaluated the effects of stimulating VTA^PL^ presynaptic fibers in the remaining rats, using bath application of the otherwise inert DREADD receptor ligand, clozapine‐N‐oxide (CNO). For electrophysiological experiments, acute PFC brain slices were prepared from the remaining DREADD‐injected rats (4–9 weeks after injection) to record pyramidal cells in L5 PL‐PFC (Fig. 3C). As expected, bath application of CNO (5 *μ*mol/L; 10 min) to stimulate Gq‐DREADD‐expressing VTA terminals reduced SFA at current injections above 0.4 nA (Fig. 3D). The increase in action potential firing at current injections 0.5–1 nA (Fig. 3E) and the reduction in the AHP (Fig. 3F) in response to CNO was uniform across cells (*n* = 13 cells/4 rats). We noted similar, but less robust effects with CNO stimulation of Gs‐DREADD expressing VTA^PL^ fibers in that SFA, but not the AHP, was significantly reduced by CNO (Gs‐DREADDs—APs: *t*_(5)_ = 2.846, *P* = 0.036 at 1 nA of injected current; AHP: *t*_(5)_ = 0.42, *P* = 0.6907). Together, these results indicate stimulation of VTA^PL^ terminals with DREADDs reduces intrinsic inhibition of pyramidal cells in the PL.

We next determined if chemogenetic stimulation of VTA^PL^ fibers altered intrinsic inhibition in PL cells innervating the NAcC. We again transfected the VTA of *TH::Cre* ^*+*^ transgenic rats with Gq‐DREADDs, and also injected fluorescently labeled beads in NAcC to retrogradely label PL neurons in this circuit (Fig. 3G). We made acute PFC brain slices from these rats, confirmed localized bead injections in the NAcC, and then recorded from retrobead‐positive PL cells identified via fluorescence (Fig. 3H). We observed that stimulation of VTA^PL^ fibers with CNO significantly reduced SFA at current injections above 0.5 nA (Fig. 3I) and the AHP integral (Fig. 3J). While these data demonstrate that VTA^PL^ terminals shape intrinsic inhibition in PFC cells projecting to the NAcC, it remains to be determined whether this is a circuit‐specific action, or whether other circuits, for example, PFC cells projecting to the nucleus accumbens shell, show similar sensitivity to VTA terminal stimulation.

Collectively, these data suggest that activation of VTA^PL^ terminals reduces intrinsic inhibition in PL cells that innervate the NAcC, a circuit known to be important for a variety of dopamine‐dependent behaviors.

## Discussion

The prefrontal cortex is important for behavioral flexibility, working memory, and cognitive control. The VTA, in general, and dopamine, in particular, have an essential role in these processes (Naneix et al., 2009). A general principle evolved from past studies is that dopamine released from VTA axons acting on D1 receptors in the prefrontal cortex (PFC) facilitates function in accordance with an “inverted U” function, until at higher concentrations activation of D2 receptors produce detrimental actions on performance. Consistent with this, electrophysiological studies in PFC cells show complex excitatory and inhibitory responses to bath applied dopamine, and PFC dopamine levels correlate with VTA dopamine cell firing patterns (Seamans et al., 2001; Seamans and Yang, 2004). Given the hypothesized role of VTA dopamine, it is perhaps surprising that there exists little evidence to support or refute the direct involvement of VTA terminals in cortical function. To the best of our knowledge, there exist no studies showing that selective activation of VTA axon terminals alters PFC neuron plasticity. To address this, we corroborated previously reported actions of bath applied dopamine, and then tested the responsiveness of similar cells to optogenetic or chemogenetic activation of TH‐positive VTA axons in PFC slices. Across these different treatments, we found remarkably similar changes in PFC neuron excitability supporting the hypothesis that VTA regulates PFC function through an action involving the release of dopamine from axon terminals.

The most important aspect of this study was the experiments showing a slow, progressive change in SFA and the AHP in PFC cells during photoactivation or chemogenetic stimulation of VTA axon terminals. Neither blue light nor CNO produced any changes in tissue from animals not transfected with virus. Yet, SFA/AHP declined reliably during the exposure to repeated phasic‐like photoactivation patterns or bath application of dopamine with cocaine, a blocker of dopamine reuptake, or CNO activation of Gq‐DREADDs. A logical interpretation of these data is that the phasic stimulation of VTA axons and blockade of dopamine reuptake or the continuous stimulation of axons with DREADDs caused a buildup of extracellular dopamine in the slice that activated dopamine receptors. This scenario would parallel results from another optogenetic study in the nucleus accumbens showing stimulation of dopamine release is both possible and efficient with similar low‐light power levels (Lu et al., 2015). Consistent with our study, that work found that increases in extracellular concentrations of dopamine release were sensitive to both changes in the light pulse width as well as the temporal pattern of pulses delivered (Lu et al., 2015).

An alternative scenario is that photo/chemostimulation of these terminals released glutamate, which increased spiking. Prior studies show a population of VTA dopamine neurons that also express vesicular glutamate transporter 2 make glutamatergic connections with regions such as the nucleus accumbens (Mingote et al., 2015). Although that study indicated dopamine neuron glutamatergic connections in the anterior cortices are very weak (Mingote et al., 2015), another study suggested a higher percentage in this general region (Gorelova et al., 2012). Our high‐power photomicrographs revealed a significant density of ChR2‐EYFP/TH and Gq‐DREADD‐mCherry/TH axons present in the PFC, but we did not systematically compare the incidence and strength of dopaminergic to glutamatergic connections across animals. So, we cannot explicitly rule out the possibility of a glutamatergic contribution, but find it less likely. Our brain slices were incubated in the irreversible NMDA receptor antagonist MK801. We also found that bath application of NBQX to block AMPA receptors did not prevent or reverse our observed changes in SFA/AHP.

Instead, our study examined the response to bath applied dopamine, and observed similar robust changes in SFA/AHP that showed increased sensitivity during bath application of cocaine. Although we did not determine the receptor subtype activated by dopamine, numerous previous studies have addressed the relevant cellular actions of dopamine D1‐ and D2‐like receptors that orchestrate an “inverted‐U” PFC response to dopamine (Trantham‐Davidson et al., 2004; Tseng et al., 2007). In brain slices, bath application of dopamine directly depolarizes PFC pyramidal cells with concentration‐dependent actions that persist in the presence of synaptic blockade (Shi et al., 1997). Consistent with this, dopamine or a D1 receptor agonists reduce spike latency and lower the firing threshold of the PFC neurons in response to depolarizing current pulses via changes attributed to sodium channels, potassium channels, and calcium spikes (Yang and Seamans, 1996b). In vivo administration of D1 receptor antagonist prevents LTP in the PFC, and result in long‐term depression‐like responses (Coppa‐Hopman et al., 2008). Conversely, PFC (L5) pyramidal cells are more sensitive to the induction of LTP induced by D1‐like dopamine receptors following a short‐term (3 days) withdrawal from noncontingent cocaine (Huang et al., 2007). Cocaine can facilitate LTP induction via sensitized D1‐cAMP/PKA dopamine signaling in pyramidal neurons (Ruan and Yao, 2017). Yet, other studies show dopamine can also decrease the number of action potentials via direct activation of D2 receptors (Gulledge and Jaffe, 1998) or indirect actions of D1 receptors that alter the synaptic release of GABA from fast‐spiking interneurons and glutamate terminals (Law‐Tho et al., 1994; Gorelova et al., 2002; Gao and Goldman‐Rakic, 2003). Most recently, a study showed that both the dopamine suppression and enhancement of PFC pyramidal neuron excitability involving dopamine D1 and D2 receptors was attributable to differential activation of intracellular cAMP–PKA signaling (Yang et al., 2013). Based on these literature, it is tempting to speculate that our findings showing increased spiking reflect activation of D1 dopamine receptors and activation of intracellular cAMP–PKA signaling. However, many of these previous studies were performed in younger aged animals, which influence D1 dopamine receptor‐mediated processes and complicate any such conclusions (Parfitt et al., 1990).

Other studies at the systems level support the functional association between D1 receptors in the PFC, dopamine terminals emanating from the VTA, and PFC projections to the nucleus accumbens. Optogenetic activation of VTA dopamine cells facilitates temporal control for some tasks via recruitment of D1‐expressing PFC cells (Narayanan et al., 2012; Land et al., 2014; Kim et al., 2017). Optogenetic inactivation of the PFC to the accumbens core pathways decrease cocaine seeking in rodent models of relapse (Stefanik et al., 2013; Martín‐García et al., 2014). Two photon imaging studies indicate that phasic dopamine neuron activity potentiates the size and plasticity of VTA axonal boutons in cortex in vivo (Mastwal et al., 2014). Bimodal actions of dopamine may help explain why prefrontal hypoactivation as well as hyperactivation (e.g., disinhibition) cause attentional deficits associated with PFC function (Pezze et al., 2014). Taken together, these literature provide support for a mechanism involving VTA axons that release dopamine in the PFC to drive a neural circuit that innervates the nucleus accumbens and regulate executive control of reward and addiction‐related behaviors (See, 2002; Kalivas and McFarland, 2003; Kalivas, 2009).

## Conclusion

These results suggest that dopamine release from VTA terminals gates intrinsic inhibition in the cortex by regulating SFA and the AHP. This plasticity was induced with burst‐like stimulation of VTA terminals, in cortical neurons that project to the accumbens core. Dopamine‐induced reductions in intrinsic inhibition were potentiated by blockade of dopamine reuptake with acute exposure to cocaine. Such a mechanism may contribute to the VTA regulation of PL‐PFC activity during the presentation of rewards and their cues.

## Conflict of Interest

None declared.
